# Proactive Effect of Algae-Based Graphene Support on the Oxygen Evolution Reaction Electrocatalytic Activity of NiFe

**DOI:** 10.3390/ma16247641

**Published:** 2023-12-14

**Authors:** María González-Ingelmo, Marcos Granda, Begoña Ruiz, Enrique Fuente, Uriel Sierra, Victoria G. Rocha, Zoraida González, Patricia Álvarez, Rosa Menéndez

**Affiliations:** 1 Instituto de Ciencia y Tecnología del Carbono (INCAR), CSIC, Francisco Pintado, Fe 26, 33011 Oviedo, Spain; maria.ingelmo@incar.csic.es (M.G.-I.); mgranda@incar.csic.es (M.G.); begorb@incar.csic.es (B.R.); enriquef@incar.csic.es (E.F.); vgarciarocha@incar.csic.es (V.G.R.); zoraidag@incar.csic.es (Z.G.); 2 Laboratorio Nacional de Materiales Grafénicos, Centro de Investigación en Química Aplicada, Blvd. Enrique Reyna Hermosillo, 140, Saltillo 25294, Mexico; uriel.sierra@ciqa.edu.mx

**Keywords:** graphene, macro-algae, water splitting, OER

## Abstract

The preparation of graphene materials from biomass resources is still a challenge, even more so if they are going to be employed as supports for electrocatalysts for water splitting. Herein, we describe the preparation and characterization of graphene oxides (GOs) from solid macroalgae waste obtained after processing an agar–agar residue. The structural and morphological characterization of the obtained GO confirm the presence of a lamellar material that is composed of few layers with an increased number of heteroatoms (including nitrogen) if compared with those observed in a GO obtained from graphite (reference). Three-dimensional electrodes were prepared from these GOs by depositing them onto a fibrous carbon paper, followed by electrodeposition of the catalyst, NiFe. The electrocatalytic performance of these hybrid systems for the oxygen evolution reaction (OER) showed a proactive effect of both graphene materials toward catalysis. Moreover, the electrode prepared from the algae-based graphene showed the highest electrocatalytic activity. This fact could be explained by the different structure of the algae-based graphene which, due to differences in the nucleation growth patterns and electroactive sites developed during the electrodeposition process, produced more reactive NiFe species (higher oxidation state).

## 1. Introduction

The increasing demand for renewable energy systems has guided researchers to look for environmentally friendly and cheaper energy sources [1,2]. The electrolysis of water has emerged as an effective approach for producing hydrogen, a clean energy source that can be used as a sustainable alternative to traditional fossil fuels [3,4]. The two basic half-reactions involved in the process are the hydrogen evolution reaction (HER) and oxygen evolution reaction (OER), with the latter exhibiting sluggish kinetics and a high overpotential, requiring the use of an electrocatalyst. In an alkaline electrolyte, nonprecious metals, such as Ni, Co, and their alloys (NiFe, CoFe, etc.), are commonly used as electrocatalysts for the OER [5]. Among them, NiFe layered double hydroxide (LDH) has been broadly regarded as the most promising candidate because of its layered and relatively open structure, abundant catalytic sites, and low cost [6]. Nevertheless, its low conductivity and limited electrochemically active surface area (ECSA) severely restrict the electron transport step, resulting in degraded electrochemical performance and thus restricting the utilization of NiFe LDH catalysts [7,8]. Additionally, one of the major challenges in OER research is obtaining cost-effective electrodes without compromising their activity or durability/long-term performance [9,10,11,12,13].

Carbon nanomaterials, such as graphenes, have been actively investigated as catalysts alone (i.e., by controlling defects) [14,15] or as proactive supports because of their outstanding properties, including electrical conductivity, mechanical stability, tunable morphology, and functionality [16]. They contribute to enhancing electrocatalyst dispersions; act as co-catalysts of metal/non-metal particles; and optimize the physicochemical properties of the catalyst, like the loading amount, electronic structure, charge transfer rate, etc. The development of graphene technologies is, however, limiting their production at a large scale, mainly due to high costs. Graphenes are usually obtained from graphites or even other fossils, such as pre-graphitic materials [17]. In recent years, driven by sustainability and renewability concerns, new green alternatives have been explored, such as different types of biomass and biomass wastes, which can be considered carbon-neutral [18]. These materials usually contain a relatively high carbon content [19] while having low cost and high availability, enabling a drastic cost reduction for graphenes.

One of the most important challenges in the production of graphene materials from biomass is ensuring their quality. The utilization of biomass resources with many structural differences among them usually leads to structural graphene inconsistencies that should be evaluated in detail [20], particularly when the graphenes are to be used for specific applications. Aspects such as the selection of the biomass and the preparation methodology are key factors to be taken into account when obtaining a graphene layer with specific characteristics.

Bearing this in mind, the first objective of this work is to evaluate agar–agar industrial waste as a raw material for the preparation of graphene oxides. This waste was obtained after processing the macroalgae *Gelidium sesquipedale* (currently called *Gelidium corneum*) generated by the largest European producer of agar–agar, located in the north of Spain. The waste was initially processed for energy use exploitation through conventional and microwave pyrolysis technologies, obtaining a solid residue (bio-char), a liquid (bio-oil), and a gas with different physicochemical properties [21,22]. The solid residue was successfully upgraded to obtain activated carbon via conventional and microwave chemical activation. Moreover, the activated carbon exhibited adequate characteristics for use in the removal of mercury present in combustion gases and in the storage of gases at high pressures [23,24,25]. However, its valorization to graphene materials has not been investigated.

Taking into account the sustainable development criteria, a new use for this macroalgae waste is proposed. Graphene oxides were prepared from the bio-char obtained through the conventional high-temperature pyrolysis of the waste. This rational use of macroalgae solid residue is an interesting proposal, representing an alternative to its use as fertilizer or fodder or its disposal in landfills.

The graphenes were produced by means of a modified Hummers method, including an additional purification step, and were fully/deeply characterized. Via this methodology, the obtained graphene oxides exhibited the oxygen functional groups required to prepare suitable 3D electrodes for OER. The electrodes were prepared by drop-casting the GOs on fibrous paper (Toray carbon paper) followed by electrodeposition of the NiFe catalyst. A detailed chemical and morphological characterization of the electrodes was conducted in order to correlate the results with the subsequent OER performance.

## 2. Materials and Methods

### 2.1. Preparation of Graphene Oxide-like Materials from Algae

Macroalgae waste (Al) was used in this study as a raw material. It was obtained after carbonizing agar–agar residue for an energy upgrade in an inert atmosphere at 1000 °C and subsequently grinding and sieving it below 75 μm. For comparative purposes, commercial graphite (G) was also used as a raw material. The graphene oxide material obtained from the macroalgae waste (Al-GO) was prepared as follows [26,27]: Al was treated with 1 M KOH, followed by 40% HNO_3_. Chemical oxidation was carried out by means of a modified Hummers method for 3 h at 35 °C in the presence of H_2_SO_4_, NaNO_3_, and KMnO_4_. After stopping the reaction with 3% H_2_O_2_, the material was washed using distilled water by centrifugation. After that, the material was exfoliated in ultrasound for 8 h, obtaining the desired GO-like material as an aqueous suspension.

The obtained graphene oxide-like materials were labeled as Al-GO (from the macroalgae waste) and G-GO (from graphite).

### 2.2. Preparation of 3D Electrodes

Toray carbon paper (TCP) (TGP-H-60), previously treated at 550 °C for 1 h in air atmosphere to improve its wettability, was used as the support for the 3D electrode. This TCP was immersed in an aqueous suspension of the corresponding graphene oxide materials (Al-GO and G-GO) (2000 ppm) for 30 min and subsequently thermally treated at 400 °C (the graphene oxides thermally treated at 400 °C were labeled Al-GO-400 and G-GO-400). The resultant films were used as working electrodes (WEs) for the subsequent electrodeposition of NiFe nanoparticles in a three-electrode electrochemical cell, with a graphite rod being used as the counter electrode (CE) and Ag/AgCl (3.5 M KCl) being used as the reference electrode (RE). The electrolyte contained an equal molar composition (3 mM) of nickel (II) and iron (III) nitrates [28]. A constant electrodeposition potential of −1.0 V (versus RE) at 10 °C was applied for 300 s. The electrode was then rinsed with water and ethanol and dried to yield the 3D electrodes TCP-Al-GO-400-NiFe and TCP-G-GO-400-NiFe.

### 2.3. Characterization of Graphene Materials and 3D Electrodes

The carbon, hydrogen, nitrogen, sulfur, and oxygen contents of the samples were determined using both a Perkin–Elmer 2400 Series-II microanalyzer (Perkin Elmer, Inc., Waltham, MA, USA) and LECO-932 microanalyzer (LECO Corporation, Groveport, OH, USA), the latter being equipped with a LECO-VTF-900 furnace (LECO Corporation, Groveport, OH, USA).

Transmission electron microscopy (TEM) investigations, including conventional (CTEM), high-resolution (HRTEM), and elemental chemical analysis (EDX) methods, were performed on graphene specimens using an FEI TITAN 80–300 kV instrument (FEI Technology de México S.A., Monterrey, Mexico). It operated at an accelerating voltage of 300 kV. Suspensions of graphene oxide in ethanol were deposited on a 200-mesh cooper grid for observations. Scan lines, areas, and specific chemical analyses were obtained using scanning transmission electron microscopy (STEM, ThermoFisher Scientific, Hillsboro, OR, USA) operating at 300 kV.

SPECS equipment (SPECS Group, SPECS Surface Nano Analysis GmbH, Berlin, Germany) operating under a pressure of 10^−7^ Pa with a Mg Kα X-ray source was used for the X-ray photoelectron spectroscopy (XPS) studies. Deconvolution of the XPS peaks was performed using a Shirley baseline and a combination of Gaussian and Lorentzian functions [29].

A Zeiss DSM 942 microscope (Carl Zeiss Iberia, S.L—Division Microscopy, Madrid, Spain) was used for the scanning electron microscope (SEM) observations.

Raman spectroscopic studies were performed using a Renishaw 2000 Confocal Raman Microprobe device (Renishaw Ibérica, Barcelona, Spain), operating at 514.5 nm in argon and covering a wavelength of 750–3500 cm^−1^.

### 2.4. Electrochemical Characterization

Electrochemical measurements were performed under ambient conditions in a custom-made Teflon three-electrode cell, with 1 M KOH being used as the electrolyte. The cell configuration included the previously prepared 3D electrodes as the WEs, an Ag/AgCl/3.5 M KCl as the RE, and a graphite rod as the CE. The electrochemical behavior of the samples was evaluated in the potential range between 1.2 and 1.8 V vs. the reversible hydrogen electrode (RHE). The potentials in this section were converted to the RHE scale for comparative purposes and calculated according to the Nernst equation: E_RHE_ = E_Ag/AgCl_ + 0.059 pH + 0.216 V. Initially, cyclic voltammetry (CV) experiments (50 mVs^−1^) were performed to stabilize the OER activity of the active materials. Linear sweep voltammetry (LSV) at 10 mVs^−1^ was then conducted, and the corresponding Tafel slope values were calculated. The catalytic activity is reported in terms of the current density in mAcm^−2^, where cm^2^ refers to the geometric exposed area of the WE (1 cm^2^). The stability of the electrodes TCP-Al-GO-400-NiFe and TCP-G-GO-400-NiFe was assessed through chronopotentiometry (CP) experiments at 10 mAcm^−2^ for 1 h. Electrochemical impedance measurements (EISs) were also carried out on these electrodes to evaluate the R_ct_ values. In the EIS experiments, a potential perturbation of 10 mV was applied within a frequency range spanning from 100 kHz to 100 mHz. The EIS measurements were acquired under a constant potential of 1.6 V vs. the RHE, the potential at which OER is assumed to occur.

## 3. Results and Discussion

### 3.1. Preparation of Graphene Materials from Algae Waste

The macroalgae raw material used in this work was generated as a residue in the industrial processing of high-quality algae of the genus *Gelidium*, generally *sesquipedale* (currently called *corneum*), to obtain agar–agar, a polysaccharide used to gel, retain water, and stabilize products in sectors such as foods, pharmaceuticals, etc. The industrial process of macroalgae transformation has previously been described [22]. Briefly, the macroalgae waste (algae meal) generated in the industrial process was subsequently pyrolized up to 1000 °C, yielding a bio-char labeled as Al, which was the parent material used here.

As determined by the XPS analysis, it was mainly composed of C and O and, to a lesser extent, by other elements inherent to its biological nature, such as N, S, K, Ca, Mg, Na, and P (Table 1).

The graphene oxide-like material from the algae-derived waste (Al-GO) was obtained by means of an optimized Hummers method. For comparative purposes, graphene oxide from commercial graphite was also prepared under the same experimental conditions (G-GO). This method includes, among other modifications (see experimental section for details), an initial acid treatment aimed to eliminate most of the non-organic elements present in its composition. The reaction time was also increased, and a lower reactant concentration was used. By means of this procedure, the yields obtained were 60 wt.% Al-GO and 70 wt.% G-GO.

An analysis of the composition of Al-GO, as determined by XPS (Table 1), indicated that this material mainly consisted of C (72.3 at.%) and O (26.4 at.%). Surprisingly, this material was less oxidized than the GO obtained from graphite (G-GO: C: 65.8%; O: 34.2%). In order to explain this fact, we propose herein that, in the algae-based material, the oxidant was mainly consumed by the non-graphitic carbonaceous structures and, consequently, lost. It is also important to reiterate that Al-GO, despite being less oxidized, contained small amounts of N (1.1 at.%) and S (0.3 at.%), which were not seen in G-GO. These elements seem to be part of their carbonaceous aromatic structure and are inherent to the biological nature of the raw material.

**Figure 1:**
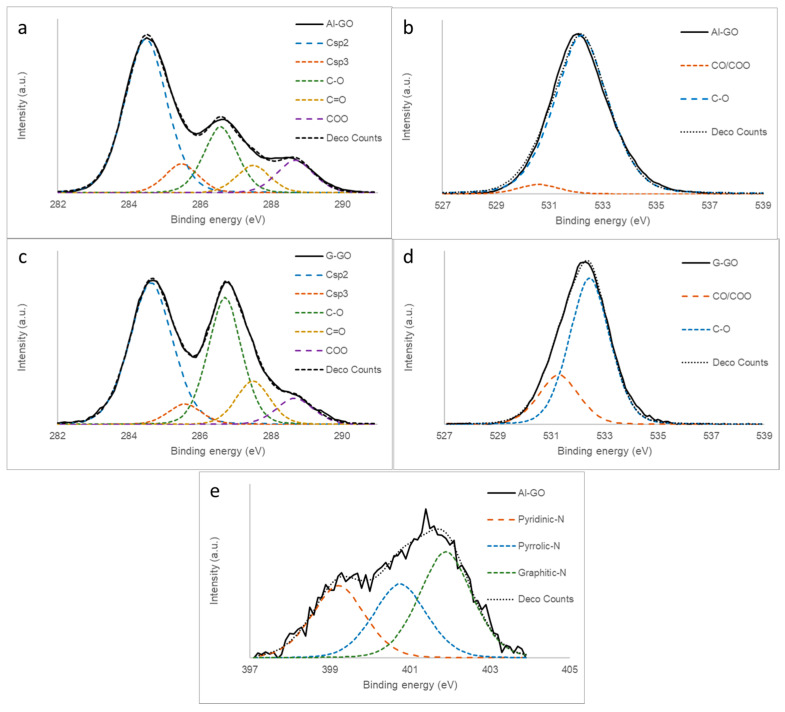
XPS spectra of (**a**) C1s of Al-GO, (**b**) O1s of Al-GO, (**c**) C1s of G-GO, (**d**) O1s of G-GO, and (**e**) N1s of Al-GO.

The high-resolution XPS C1s spectra of Al-GO, G-GO, and their deconvolutions confirm our previous results (Figure 1a,c). G-GO exhibited a bimodal C1s curve typical of graphene oxide [17,26,29,30], with two main peaks ascribed to Csp^2^ carbons (284.5 eV) and C-O carbons (286.5 eV). However, the C-O peak in Al-GO was significantly less intense, as the majority of the carbon atoms were sp^2^-hybridized, corresponding to a poorly oxidized graphene material. The O1s XPS spectra of Al-GO and G-GO were quite similar (Figure 1b,d) and corresponded to the typical shape of a GO, exhibiting a main wide band at 532.5 eV (possibly C-O with a higher contribution of epoxy groups) with a small contribution of CO/COO groups (530.5 eV) [30]. On the other hand, the N1s spectra of Al-GO, although of low intensity, suggested a heterogeneous distribution of functional groups in which pyridinic (399.5 eV) and substitutional nitrogen (401.3 eV) were present, together with a lower amount of pyrrolic nitrogen (400.5 eV) (Figure 1e) [31].

**Figure 2:**
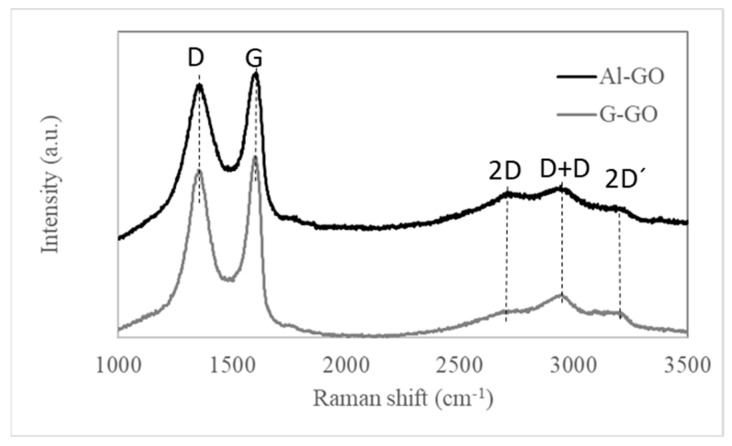
Raman spectra of Al-GO and G-GO.

The Raman spectrum of Al-GO has the typical shape of graphene oxide, very similar to that of G-GO (Figure 2) [32]. They both exhibited a first-order Raman region (below 2000 cm^−1^) in which two main peaks could be observed at 1365 cm^−1^ (D peak, ascribed at the breathing modes of sp^2^ rings and directly connected with the number of defects) [20,33] and 1605 cm^−1^ (G band, related to the quality and degree of graphitization) [34]. Additional weak peaks at 1130 and 1770 cm^−1^ were detected, which were observed in other graphene oxides [32]. The I_D_/I_G_ ratio obtained for Al-GO was 0.95, slightly lower than that calculated for G-GO (0.97). The spectra also show the second-order Raman peaks, with the appearance of three peaks: 2D, D+D, and 2D′ at 2715, 2959, and 3211 cm^−1^, respectively, which is typical of graphene oxide structures [14,28,29].

**Figure 3:**
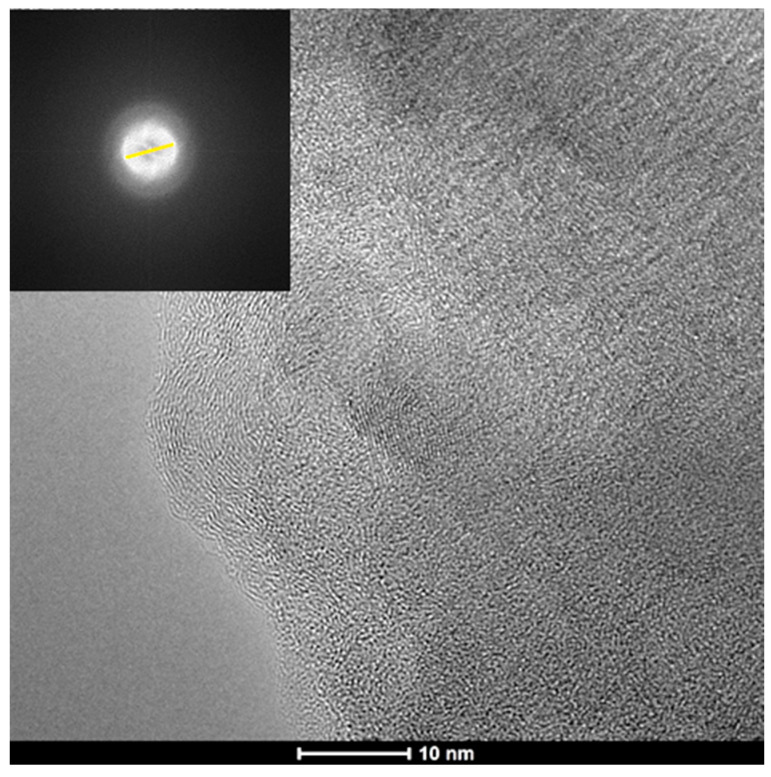
High-resolution transmission electron microscopy (HRTEM) and FFT images of Al-GO.

The sheet-like structure of Al-GO was confirmed by an HRTEM analysis (Figure 3) and was mainly in the form of a few-layer graphene oxide material. The appearance of wrinkles and defective edges was also noticed, similarly to other graphene oxide sheets from biomass sources [35,36]. The fast Fourier transformation (FFT) pattern, shown in the Figure 3 inset, indicates the presence of a polycrystalline structure corresponding to what is expected for a graphene oxide [37,38,39]. The interlaminar distance corresponds to the d = 0.34 nm, which is typical of graphitic materials.

The previous results confirm the presence of laminar carbonaceous materials, which match well with that of a graphene oxide, thus confirming its formation from an algae-based residue. All of these results confirm the possibility of preparing a graphene oxide-like structure from algae-derived material.

### 3.2. Synthesis, Characterization, and Evaluation of the Catalytic Activity of Algae-Based and Graphite-Based 3D Electrodes

The first step in the preparation of the 3D-electrodes was the drop-casting of the graphene materials onto the TCP, followed by thermal reduction to 400 °C. This temperature was optimized to yield electrodes with the highest catalytic OER activity. As can be seen in Figure S1 (see Supplementary Materials), a higher temperature (800 °C) was tested for the graphite-derived electrode; no improved catalytic activity was obtained.

**Figure 4:**
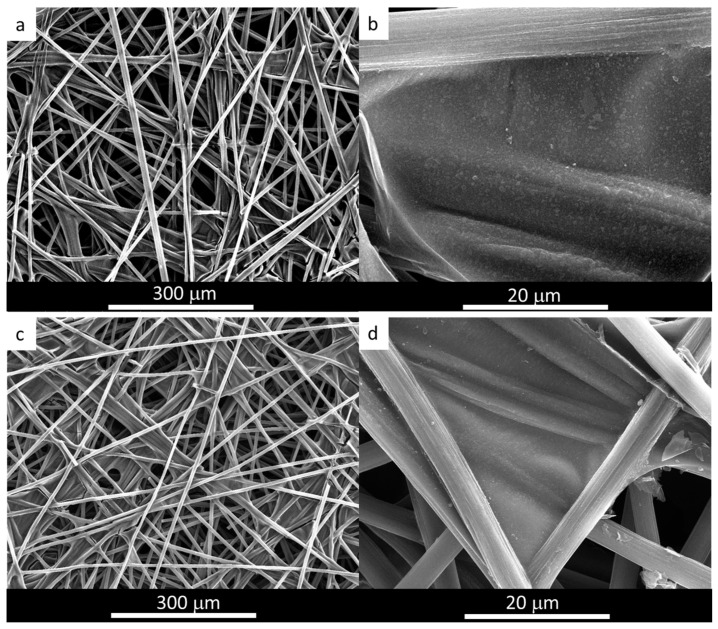
SEM analysis of algae-based graphene material (**a**,**b**) deposited on TCP and (**c**,**d**) after electrodeposition of NiFe.

The materials were analyzed by SEM, and the resultant images were compared with that of bare TCP SEM images (Figure S2). As a result of the processing, thin sheets of graphene material were attached to the TCP fibers, recovering all of them and also being located at their joints, as determined by an SEM analysis for the algae-derived electrodes (Figure 4). Moreover, the films prepared from the two GOs used in this work showed no substantial differences in their morphology (see Supporting Information, Figure S3).

**Figure 5:**
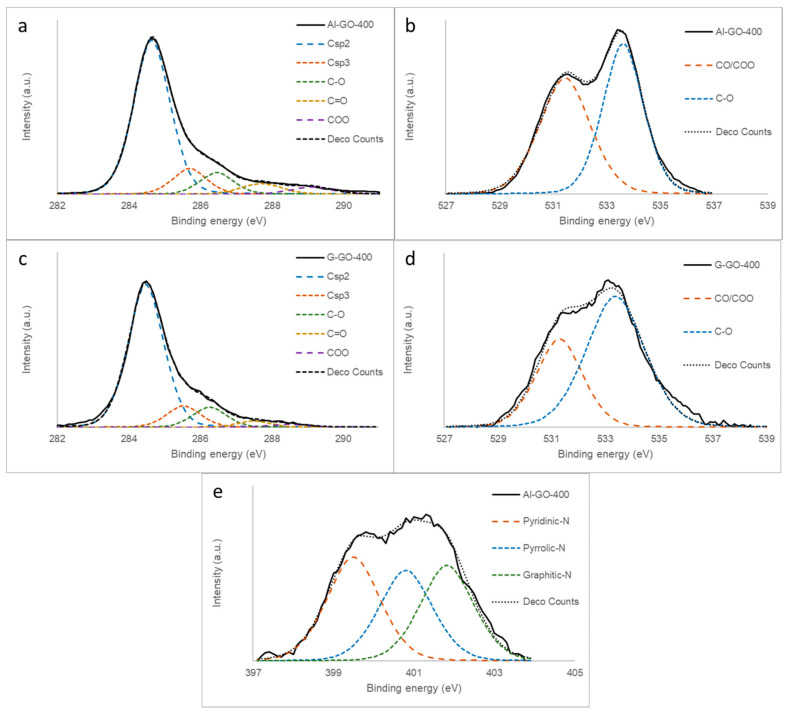
XPS spectra of (**a**) C1s of Al-GO-400 (**b**) O1s of Al-GO-400, (**c**) C1s of G-GO-400, (**d**) O1s of G-GO-400, and (**e**) N1s of Al-GO-400.

The thermal step was required in order to increase the stability of the TCP/graphene system (ensuring an appropriate contact between carbon fibers and graphene sheets) and to increase the electrical conductivity of the graphene material. This was achieved by removing the oxygen functional groups from the graphene sheet, with the temperature, as confirmed by the XPS analysis of the thermally treated graphenes, being at 400 °C (Table 1). The Csp^2^ network was also reconstructed, as observed by the XPS C1s analysis (Figure 5a,c). This reconstruction proceeded to a lesser extent in Al-GO-400, as its precursor, Al-GO, was less oxidized. Such structural thermal evolution contributed to decreasing the differences between both thermally reduced graphene materials prepared from graphite and algae waste; moreover, both reduced graphene oxides exhibited similar shapes to the XPS C1s spectra (Figure 5a,c). The evolution of the oxygen functional groups was also evident in the O1s spectra (Figure 5b,d), which became a wider bimodal curve, as expected for thermally reduced graphene oxides [32]. More importantly, the nitrogen content of Al-GO-400 was even higher after the thermal treatment. Moreover, the N1s spectra of this sample (Figure 5e) was quite similar to that of the Al-GO graphene (Figure 1e) without thermal reduction. This result suggests that the nitrogen present in the algae-based GO (natural nitrogen) is thermally stable nitrogen within the carbonaceous structure of the graphene sheet.

**Figure 6:**
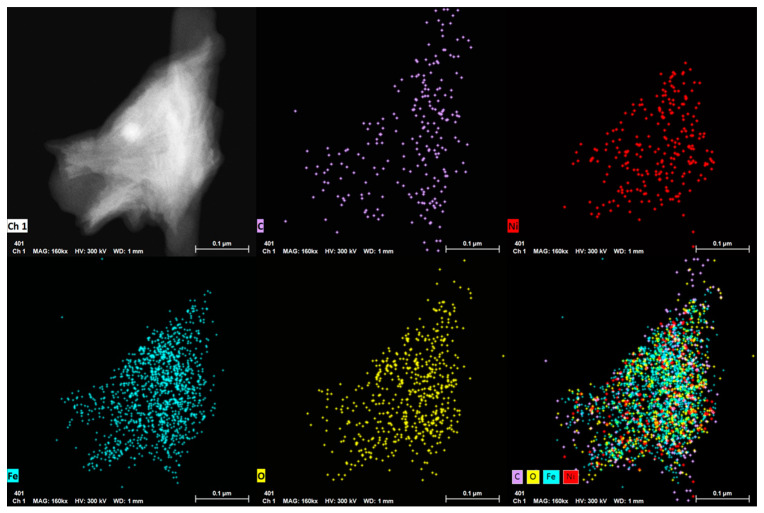
TEM-EDX images and mapping of TCP-Al-GO-400-NiFe.

The morphology of the TCP/graphene 3D electrode after the electrodeposition of NiFe, as determined by SEM, remained unaltered (Figure 4c,d for TCP-Al-GO-400-NiFe), suggesting the formation of a thin film phase of NiFe material homogeneously distributed on the surface of the graphene film. This result is similar to that observed for TCP-G-GO-400-NiFe (Figure S3). Moreover, the analysis of this NiFe film was conducted by a TEM analysis of the peel-off layers. The results for the algae-derived materials TCP-Al-GO-400-NiFe showed that the electron-dense regions of the NiFe were homogeneously distributed along the carbonaceous materials, as revealed by the TEM-EDX mappings (Figure 6). Similar results were observed for TCP-G-GO-400-NiFe (Figure S5). These results confirm the homogeneous distribution of elemental Ni, Fe, and O along the graphene’s surface.

**Figure 7:**
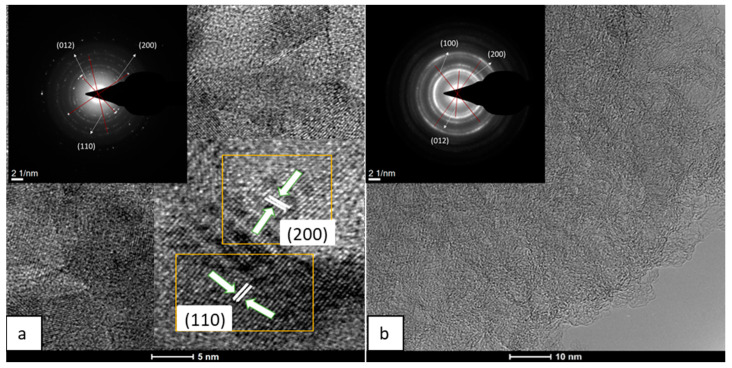
HRTEM of 3D electrodes from Al-GO (**a**) and G-GO (**b**) (TCP-Al-GO-400-NiFe (**a**) and TCP-G-GO-400-NiFe (**b**)). Insets correspond to SAEDs.

However, the HRTEM characterization of both electrodes revealed some structural differences between them (Figure 7). The selected area electron diffraction pattern (SAED) analysis of the algae-based material (Figure 7a) showed short-range ordered regions. The distinct lattice spacing was 0.15, 0.17, and 0.25 nm, corresponding to the (100), (200), and (012) plane of the NiFe LDH [40]. In the analysis of TCP-G-GO-NiFe, the crystalline phase of the graphene was more visible in the SAED spectra (Figure 7b and inset), with a series of concentric polycrystalline rings corresponding to the graphene-based support together with the crystalline facets of the NiFe LDH. Moreover, a detailed analysis of the SAED revealed the presence of the same distinct lattice spacing of planes (100), (200), and (012) of the NiFe LDH as in the case of TCP-Al-GO-NiFe, confirming the presence of the NiFe LDH phase in both samples.

**Figure 8:**
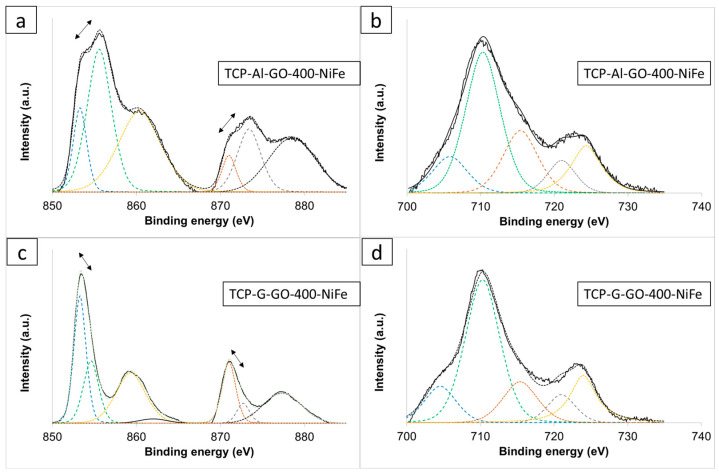
XPS spectra (solid lines) and deconvolution curves (dashed colorful lines) of (**a**) Ni2p of TCP-Al-GO-400-NiFe, (**b**) Fe2p of TCP-Al-GO-400-NiFe, (**c**) Ni2p of TCP-G-GO-400-NiFe, and (**d**) Fe2p of TCP-G-GO-400-NiFe.

To gain a deep knowledge into the structural composition of each 3D electrode, they were analyzed using XPS (Figure 8). The Fe2p spectra of both electrodes (Figure 8b,d) showed peaks, including a small metallic Fe peak at 704.9 eV (possibly as a consequence of the reduction in Fe^3+^ at the applied negative potential of −1.0 V) [41] and two spin–orbit doublet peaks located at ~712 eV and ~726 eV. The satellites’ peaks were located at ~718 eV and ~734 eV, belonging to Fe^3+^. No significant differences were observed in either electrode. In contrast, the Ni 2p_3/2_ spectra of both electrodes showed some peculiarities (Figure 8a,c). Both exhibited two sets of spin–orbit doublets and two sets of satellites. The first doublet at ∼853 and ∼871 eV and the second doublet at ∼855 and ∼873 eV were assigned to Ni^2+^ and Ni^3+^, respectively [42,43]. Two intensive shake-up satellites (∼861.0 and ∼879.6 eV) are usually observed for paramagnetic Ni^2+^ and arise from charge transfer multi-electron transitions [44,45]. It is important to remark on the different positions of the maximum in the two samples. The algae-derived electrode exhibited higher intensity in the peaks corresponding to the higher oxidation step (Ni^3+^), while in the TCP-G-GO-400-NiFe, the maxima were located in the doublets corresponding to Ni^2+^. Because the NiFe in both electrodes was deposited under the same experimental conditions, it seems that the structural differences found between both graphene supports play an important role. A possible explanation lies in the different morphology and chemical surface composition of the graphene supports, with a higher number of O and N groups in the case of the algae-derived GO. This could lead to differences in the nucleation growth patterns and resulting active species formed during the electrodeposition process in a way that is similar to what is observed when electrodepositing NiFe by utilizing different anions in the process [46,47].

**Figure 9:**
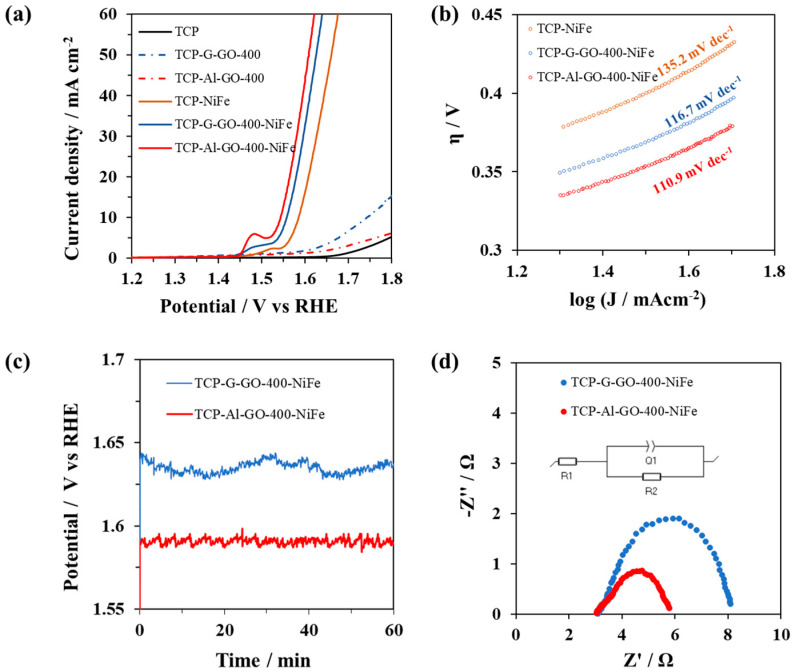
(**a**) LSVs recorded on the different electrodes under evaluation at 10 mVs^−1^ in the potential range of 1.2–1.8 V vs. the RHE and the corresponding Tafel slope values (**b**). (**c**) CPs recorded at 10 mAcm^−2^ for 1 h. (**d**) Nyquist plots collected in the frequency range between 100 kHz and 100 mHz at 1.6 V vs. the RHE. All experiments were carried out using N_2_-saturated 1 M KOH electrolytes.

The as-prepared electrodes, TCP-Al-GO-400-NiFe and TCP-G-GO-400-NiFe, were assessed for the OERs by means of CV and LSV measurements (Figure 9a). Additional studies with bare TCP (blank experiment), a TCP electrode without the presence of graphene (TCP-NiFe), and TCP in which both graphenes were deposited (TCP-Al-GO-400 and TCP-G-GO-400) were also conducted for comparative purposes. The analysis indicated that the intensity shown by the base TCP as well as by the samples in which graphene oxide was deposited (TCP-Al-GO-400 and TCP-G-GO-400) was very low, with potentials below 1.7 eV. On the one hand, this suggests a low carbon corrosion of TCP at the potentials under study. On the other hand, this suggests that neither graphene showed intrinsic catalytic activity at the levels of potential under study, as can be seen in other graphene-based materials [14,15]. This is even the case for defective and nitrogen-doped graphene (algae-based graphene). It is, however, interesting to compare the activity of the prepared hybrid materials with that of TCP-NiFe. The results indicate that the presence of graphene in both cases of 3D NiFe electrodes (TCP-Al-GO-400-NiFe and TCP-G-GO-400-NiFe) led to lower overpotentials than that measured for the TCP-NiFe electrode. This suggests a proactive effect of the graphene on the OER catalysis when used as catalyst support. Even more interesting, there was an improvement observed in the catalytic performance when using the electrode containing algae-based graphene (TCP-Al-GO-400-NiFe) compared with that containing graphene from graphite (TCP-G-GO-400-NiFe). The overpotential at 10 mAcm^−2^ for TCP-Al-GO-400-NiFe was about 310 mV, a value comparable with other NiFe composites [48] and about 20 mV lower than that obtained with TCP-G-GO-NiFe (330 mV). The excellent OER activity of TCP-Al-GO-400-NiFe was further confirmed by the smaller Tafel slope calculated, i.e., 110.9 mVdec^−1^ (Figure 9b). The presence of nitrogen in the graphene material, as well as the presence of a larger number of defects in its graphenic layer, seem to play important proactive roles in the OER catalytic cycle in a similar way to that observed for metal-free carbon nanomaterials [14,15].

Both 3D electrodes also exhibited outstanding long-term stability, as measured by CP experiments at 10 mAcm^−2^ (Figure 9c), with no signal of detachment or shrinking of the NiFe catalysts deposited on the graphene materials. Moreover, after one hour of the experiment, the potential required to achieve the set current density remained constant. Cyclic voltammetries were performed before and after the previously discussed stability test (10 mAcm^−2^), with neither hybrid material observing any substantial differences from the initial analysis (Figure S6). In addition, the SEM analysis conducted for the hybrid materials after the stability tests (Figure S4) showed no changes in morphology/structure for any of the materials analyzed, which suggests no structural changes or even oxidative etching occurring during the catalytic cycles under the conditions studied.

Electrochemical impedance spectroscopy (EIS) was employed to further evaluate the electron transfer kinetics of the 3D electrodes [49]. The Nyquist plots of TCP-Al-GO-400-NiFe and TCP-G-GO-400-NiFe are shown in Figure 9d. The diameters of the semicircles developed at the high-frequency range are correlated to the active materials’ charge transfer resistance (R_ct_), and they were fitted by following the equivalent circuit described in the inset of Figure 9d. In correlation with the previous results, the impedance of TCP-Al-GO-400-NiFe was lower (2.365 Ω) than that of TCP-G-GO-400-NiFe (5.146 Ω), evidencing its better electron transfer rate.

A possible explanation for the better results in terms of the overpotential measured on TCP-Al-GO-400-NiFe with respect to that of TCP-Al-GO-400-NiFe could lie in the presence of nitrogen, as well as the more defective graphenic structure in the GO from algae resulting from its biological nature. This different structure could enhance the interaction of the graphene surface with the NiFe catalyst, leading to an even more pronounced proactive effect on its OER catalytic activity. These promising results evidence the possibility of using algae-derived waste and transforming it into a high-added value carbon material with outstanding application in green water splitting.

## 4. Conclusions

Graphene oxide (GO) was successfully prepared from an algae-based waste from the industrial processing of red *Gelidium* algae for agar–agar production.

The obtained GO exhibited structural characteristics similar to those of a GO obtained from graphite under the same experimental conditions. The difference lay in the higher number of thermally stable nitrogen groups in its carbonaceous structure as a result of its biological origin.

Three-dimensional electrodes were prepared by deposition of the algae-based GOs on a carbon fiber paper support, followed by electrodeposition of the NiFe catalysts. The electrocatalytic performance of these hybrid electrodes as catalysts of an oxygen evolution reaction (OER) demonstrated the proactive effect of the graphene support in both electrodes as well as the outstanding performance of the graphene produced from the algae waste.

A possible explanation lies in the peculiarities of the algae-based graphene support with a higher presence of nitrogen in its layer as well as a more defective graphenic structure. This allows for a deeper interaction with the supported catalyst and enhances its proactive effect on the OER reaction.

## Figures and Tables

**Table 1:** Surface chemical composition (at.%) of raw algae-based residue (Al), commercial graphite (G), and its derived graphene materials, as determined by an XPS analysis.

Sample	C (%)	O (%)	N (%)	Mg (%)	Ca (%)	Na (%)	S (%)	P (%)	K (%)
Al	72.4	18.2	1.0	0.4	3.4	0.5	1.3	0.7	1.9
Al-GO	72.3	26.4	1.1	-	-	-	0.3	-	-
Al-GO-400	84.8	13.7	1.5	-	-	-	-	-	-
G-GO	65.8	34.2	-	-	-	-	-	-	-
G-GO-400	85.9	14.1	-	-	-	-	-	-	-

## Data Availability

Data are contained within the article and supplementary materials.

## Supplementary Materials

The following supporting information can be downloaded at: https://www.mdpi.com/article/10.3390/ma16247641/s1, Figure S1: Linear sweep voltammetry of the samples TCP-G-GO-400-NiFe (blue) and TCP-G-GO-800-NiFe (green) recorded at 10 mVs^−1^ in N_2_-saturated 1 M KOH electrolyte. Figure S2: SEM image of thermally treated Toray carbon paper. Figure S3: SEM analysis of graphite-based graphene material (a,b) deposited on TCP; (c,d) after electrodeposition of NiFe. Figure S4: SEM analysis of post-catalysis hybrid electrodes (a,c) TCP-Al-GO-NiFe and (b,d) TCP-G-GO-NiFe. Figure S5: STEM-EDX images and mapping of TCP-G-GO-400-NiFe and the homogeneous distributions of elemental Ni, Fe, and O along the graphene surface. Figure S6: Potential cycling stability results for TCP-G-GO-NiFe (left) and TCP-Al-GO-NiFe (right). All experiments were carried out at 20 mVs^−1^ in 1 M KOH electrolyte.

## References

[1] Alfani D., Binotti M., Macchi E., Silva P., Astolfis M. (2021). CO_2_ power plants for waste heat recovery: Design optimization and part-load operation strategies. Appl. Therm. Eng..

[2] Huang H., Liu M., Li X., Guo X., Wang T., Li S., Lei H. (2022). Numerical simulation and visualization study of a new tapered-slope serpentine flow field in proton exchange membrane fuel cell. Energy.

[3] Jiao Y., Zheng Y., Jaroniec M., Qiao S.Z. (2015). Design of Electrocatalysts for Oxygen- and Hy-drogen-Involving Energy Conversion Reactions. Chem. Soc. Rev..

[4] Li L., Wang P., Shao Q., Huang X. (2021). Recent Progress in Advanced Electrocatalyst Design for Acidic Oxygen Evolution Reaction. Adv. Mater..

[5] Dionigi F., Zeng Z., Sinev I., Mezdorf T., Deshpande S., Bernal Lopez M., Kunze S., Zegkinoglou I., Sarodnik H., Fan D. (2020). In-situ structure and catalytic mechanism of NiFe and CoFe layered double hydroxides during oxygen evolution. Nat. Commun..

[6] Doyle R.L., Godwin I.J., Brandon M.P., Lyons M.E.G. (2013). Redox and electrochemical water splitting catalytic properties of hydrated metal oxide modified electrodes. Phys. Chem. Chem. Phys..

[7] Gong M., Dai H. (2015). A mini review on NiFe-based materials as highly active oxygen evolution reaction electrocatalysts. Nano Res..

[8] Wang Q., Shang L., Shi R., Zhang X., Zhao Y., Waterhouse G.I.N., Wu L.Z., Tung C.H., Zhang T. (2017). NiFe layered double hydroxide nanoparticles on Co,N-codoped carbon nanoframes as efficient bifunctional catalysts for rechargeable Zinc-Air batteries. Adv. Energy Mater..

[9] Meng C., Ling T., Ma T.-Y., Wang H., Hu Z., Zhou Y., Mao J., Du X.-W., Jaroniec M., Qiao S.-Z. (2017). Atomically and Electronically Couples Pt and CoO Hybrid nanocatalysts for Enhanced Electrocatalytic Performance. Adv. Mater..

[10] Tahir M., Mahmood N., Zhu J., Mahmood A., Butt F.K., Rizwan S., Aslam I., Tanveer M., Idrees F., Shakir I. (2015). One Dimensional Graphitic Carbon Nitrides as effective Metal-Free Oxygen Reduction Catalysts. Sci. Rep..

[11] Gong M., Li Y., Wang H., Liang Y., Wu J.Z., Zhou J., Wang J., Regier T., Wei F., Dai H. (2013). An Advanced Ni-Fe Layered Double Hydroxide Electrocatalyst for Water Oxidation. J. Am. Chem. Soc..

[12] Xia Z. (2016). Hydrogen evolution: Guiding principles. Nat. Energy.

[13] Tahir M., Pan L., Idrees F., Zhang X., Wang L., Zou J., Wang Z.L. (2017). Electrocatalytic oxygen evolution reaction for energy conversion and storage: A comprehensive review. Nano Energy.

[14] Jia Y., Yao X. (2023). Defects in Carbon-Based Materials for Electrocatalysis. Acc. Chem. Res..

[15] Guo K., Li N., Bao L., Zhang P., Lu X. (2023). Intrinsic carbon structural imperfections for enhancing energy conversion electrocatalysts. Chem. Eng. J..

[16] Shi Q., Zhu C., Du D., Lin Y. (2019). Robust noble metal-based electrocatalysts for oxygen evolution reaction. Chem. Soc. Rev..

[17] Sierra U., Álvarez P., Blanco C., Granda M., Santamaría R., Menéndez R. (2015). New alternatives to graphite for producing Graphene materials. Carbon.

[18] Azwar E., Mahari W., Chuah J., Vo D., Ma N., Lam W., Lam S. (2018). Transformation of biomass into carbon nanofiber for supercapacitor application—A review. Int. J. Hydrogen Enery.

[19] Zhang L., Xu C., Champagne P. (2010). Overview of recent advances in thermos-chemical conversion of biomass. Energy Convers. Manag..

[20] Athanasiou M., Yannopoulos S., Ioannides T. (2022). Biomass-derived graphene-like materials as active electrodes for supercapacitor applications: A critical review. Chem. Eng. J..

[21] Ferrera-Lorenzo N., Fuente E., Bermúdez J.M., Suárez-Ruiz I., Ruiz B. (2014). Conventional and microwave pyrolysis of a macroalgae waste from the Agar-Agar industry. Prospects for bio-fuel production. Bioresour. Technol..

[22] Cassiani-Cassiani D., Meza-González D.A., González-Delgado A.D. (2018). Environmental Evaluation of Agar Production from Macroalgae *Gracilaria* sp.. Chem. Eng. Trans..

[23] Lopez-Anton M.A., Ferrera-Lorenzo N., Fuente E., Díaz-Somoano M., Suarez-Ruíz I., Martínez-Tarazona M.R., Ruiz B. (2015). Impact of oxy-fuel combustion gases on mercury retention in activated carbons from a macroalgae waste: Effect of wáter. Chemosphere.

[24] Ferrera-Lorenzo N., Fuente E., Suárez-Ruiz I., Ruiz B. (2014). Sustainable activated carbons of macroalgae waste from the Agar-Agar industry. Prospects as adsorbent for gas storage at high pressures. Chem. Eng. J..

[25] Ferrera-Lorenzo N., Fuente E., Suárez-Ruiz I., Ruiz B. (2014). KOH activated carbon from conventional and microwave heating system of a macroalgae waste from the Agar-Agar industry. Fuel Process. Technol..

[26] Sanchez-Page B., Pérez-Mas A., González-Ingelmo M., Fernández L., González Z., Jiménez M.V., Pérez-Torrente J.J., Blasco J., Subías G., Álvarez P. (2020). Influence of graphene sheet properties as supports of iridium-based N-heterocyclic carbene hybrid materials for water oxidation electrocatalysis. J. Organomet. Chem..

[27] Hummers W.S., Offeman R.E. (1958). Preparation of Graphitic Oxide. J. Am. Chem. Soc..

[28] Lu X., Zhao C. (2015). Electrodeposition of hierarchically structure three-dimensional nickel-iron electrodes for efficient oxygen evolution at high current densities. Nat. Commun..

[29] Sherwood P.M.A., Briggs D., Seah M.P. (1990). Practical Surface Analysis in Auger and X-ray Photoelectron Spectroscopy.

[30] Yang D., Velamakanni A., Bozoklu G., Park S., Stoller M., Piner R.D., Stankovich S., Jung I., Field D.A., Ventrice C.A. (2009). Chemical analysis of graphene oxide films after heat and chemical treatments by X-ray photoelectron and Micro-Raman spectroscopy. Carbon.

[31] Deng D., Pan X., Yu L., Cui Y., Jiang Y., Qi J., Li W.-X., Fu Q., Ma X., Xue Q. (2011). Toward N-Doped Graphene via Solvothermal Synthesis. Chem. Mater..

[32] Díez Betriu X., Álvarez García S., Botas C., Álvarez P., Sánchez Marcos J., Prieto C., Menéndez R., de Andrés A. (2013). Raman spectroscopy for the study of reduction mechanisms and optimization of conductivity in graphene oxide thin films. J. Mater. Chem. C.

[33] Romano V., Torrisi L., Cutroneo M., Havranek V., Angelo G.D. (2018). Raman investigation of laser-induced structural defects of graphite oxide films. EPJ Web Conf..

[34] Ferrari A.C., Meyer J.C., Scardaci V., Casiraghi C., Lazzeri M., Mauri F., Piscanec S., Jiang D., Novoselov K.S., Roth S. (2006). The Raman fingerprint of graphene. Phys. Rev. Lett..

[35] Bykkam S., Rao V., Chakra C.H.C., Thunugunta T. (2013). Synthesis and characterization of graphene oxide and its antimicrobial activity against klebseilla and staphylococus. Int. J. Adv. Biotechnol. Res..

[36] Somanathan T., Prasad K., Ostrikov K., Saravanan A., Krishna V. (2015). Graphene oxide synthesis from Agro Waste. Nanomaterials.

[37] Roy M., Kusurkar T.S., Maurya S.K., Meena S.K., Singh S.K., Sethy N., Bhargava K., Sharma R.K., Goswami D., Sarkar S. (2014). Graphene oxide from silk cocoon: A novel magnetic fluorophore for multi-photon imaging. 3 Biotech.

[38] Bo Z., Shuai X., Mao S., Yang H., Qian J., Chen J., Yan J., Cen K. (2014). Green preparation of reduced graphene oxide for sensing and energy storage applications. Sci. Rep..

[39] Mao S., Pu H., Chen J. (2012). Graphene oxide and its reduction: Modeling and experimental progress. RSC Adv..

[40] Kumar M., Sasikumar M., Arulraj A., Rajasudha V., Murugadoss G., Kumar M.R., Peera S.G., Mangalaraja R.V. (2022). NiFe Layered Double Hydroxide Electrocatalyst Prepared via an Electrochemical Deposition Method for the Oxygen Evolution Reaction. Catalysts.

[41] Gultom N.S., Abdullah H., Hsu C., Kuo D. (2021). Activating nickel iron layer double hydroxide for alkaline hydrogen evolution reaction and overall water splitting by electrodepositing nickel hydroxide. Chem. Eng. J..

[42] Tsoukalou A., Imtiaz Q., Kim S.M., Abdala P.M., Yoon S., Muller C.R. (2016). Dry-reforming of methane over bimetallic Ni–M/La_2_O_3_ (M = Co, Fe): The effect of the rate of La_2_O_2_CO_3_ formation and phase stability on the catalytic activity and stability. J. Catal..

[43] Yi Y., Zhang P., Qin Z., Yu C., Li W., Qin Q., Li B., Fan M., Liang X., Dong L. (2018). Low temperature CO oxidation catalysed by flower-like Ni–Co–O: How physicochemical properties influence catalytic performance. RSC Adv..

[44] Kosova N.V., Devyatkina E.T., Kaichev V.V. (2007). Mixed layered Ni–Mn–Co hydroxides: Crystal structure, electronic state of ions, and thermal decomposition. J. Power Sources.

[45] Salagre P., Fierro J.L.G., Medina F., Sueiras J.E. (1996). Characterization of nickel species on several g-alumina supported nicke samples. J. Mol. Catal. A Chem..

[46] Yan Z., Sun H., Chen X., Liu H., Zhao Y., Li H., Xie W., Cheng F. (2018). Anion insertion enhanced electrodeposition of robust metal hydroxide/oxide electrodes for oxygen evolution. Nat. Commun..

[47] Wei Z., Guo M., Zhang Q. (2023). Scalable electrodeposition of NiFe-based electrocatalysts with self-evolving multi-vacancies for high-performance industrial water electrolysis. Appl. Catal. B.

[48] Li R., Xu J., Pan Q., Ba J., Tang T., Luo W. (2019). One-Step Synthesis of NiFe Layered Double Hydroxide Nanosheet Array/N-Doped Graphite Foam Electrodes for Oxygen Evolution Reactions. ChemistryOpen.

[49] Jia D., Gao H.Y., Xing L.W., Chen X., Dong W.J., Huang X.B., Wang G. (2019). 3D Self-Supported Porous NiO@NiMoO_4_ Core–Shell Nanosheets for Highly Efficient Oxygen Evolution Reaction. Inorg. Chem..

