# Supplementary material for: Proactive Effect of Algae-Based Graphene Support on the Oxygen Evolution Reaction Electrocatalytic Activity of NiFe
Source: Materials (Basel). 2023 Dec 14;16(24):7641. doi: 10.3390/ma16247641 (PMC10744590; doi:10.3390/ma16247641)
Supplement: Supplementary file 1 [file materials-16-07641-s001.zip › materials-2697382-supplementary.pdf]

Supplementary Information

Proactive Effect of Algae-Based Graphene Support on the Oxygen Evolution Reaction Electrocatalytic Activity of NiFe

María González-Ingelmo [1], Marcos Granda [1], Begoña Ruiz [1], Enrique Fuente [1], Uriel Sierra [2], Victoria G. Rocha [1], Zoraida González [1], Patricia Álvarez [1,*] and Rosa Menéndez [1,*]

[1] Instituto de Ciencia y Tecnología del Carbono (INCAR), CSIC, Francisco Pintado, Fe 26, 33011 Oviedo, Spain; maria.ingelmo@incar.csic.es (M.G.-I.); mgranda@incar.csic.es (M.G.); begorb@incar.csic.es (B.R.); enriquef@incar.csic.es (E.F.); vgarciarocha@incar.csic.es (V.G.R.); zoraidag@incar.csic.es (Z.G.)

[2] Laboratorio Nacional de Materiales Grafénicos, Centro de Investigación en Química Aplicada,
Blvd. Enrique Reyna Hermosillo, 140, Saltillo 25294, Mexico; uriel.sierra@ciqa.edu.mx

* Correspondence: par@incar.csic.es (P.Á.); rosmenen@incar.csic.es (R.M.); Tel.: +34-985119090 (P.Á. & R.M.)

1-Comparison of the reduction temperature in the OER activity.

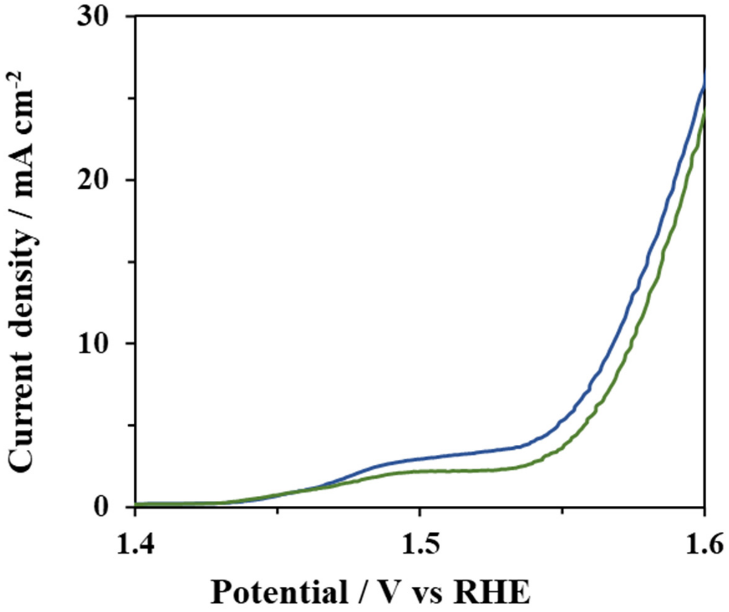

Figure S1. Linear sweep voltammetry of the samples TCP-G-GO-400-NiFe (blue) and TCP-G-GO-800-NiFe (green) recorded at 10 mVs^{-1} in N_2 saturated KOH 1 M electrolyte.

2-SEM images:

a-SEM image of base TCP

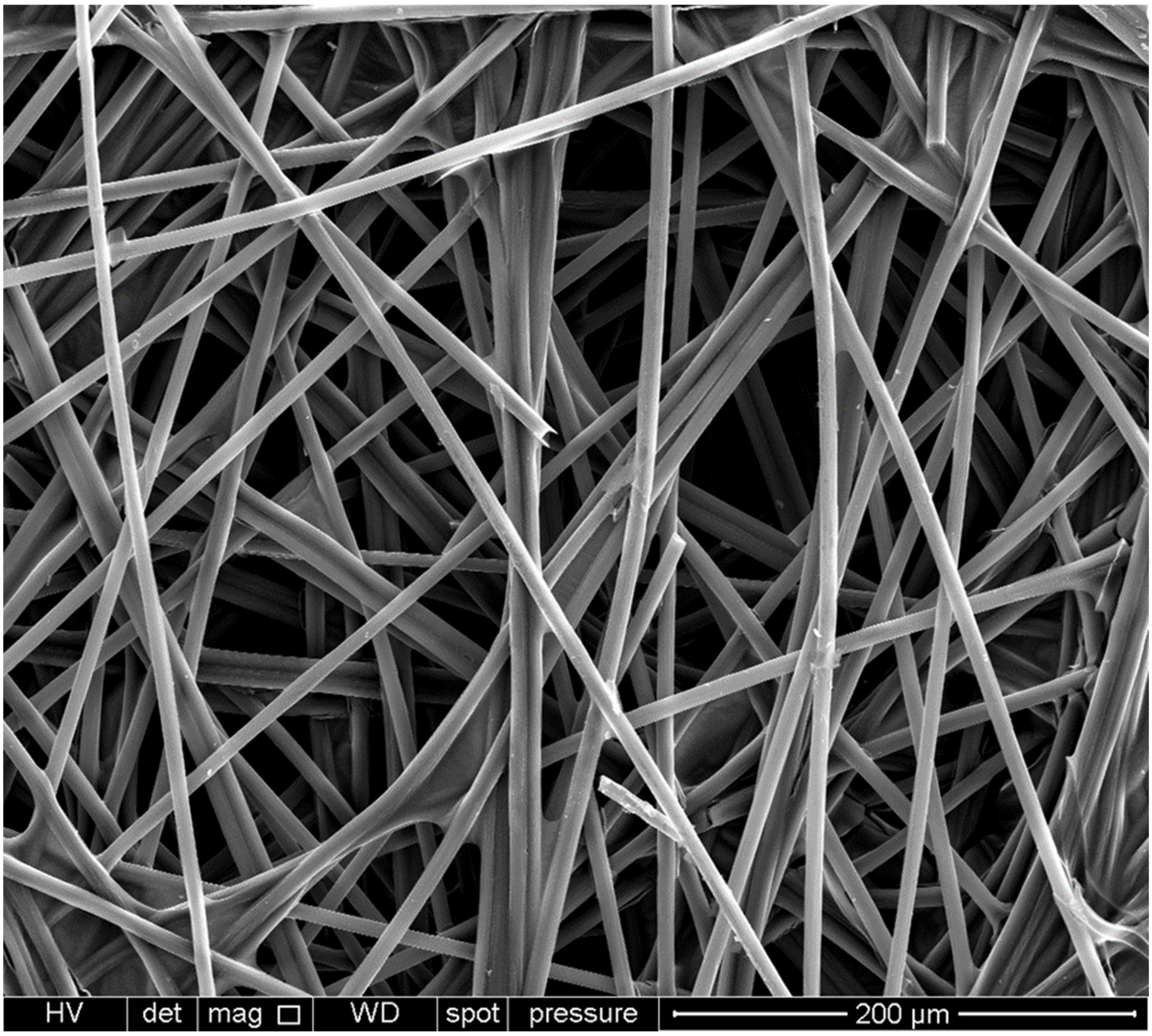

Figure S2. SEM image of thermally treated Toray Carbon Paper.

b-SEM analysis of graphite-based graphene material

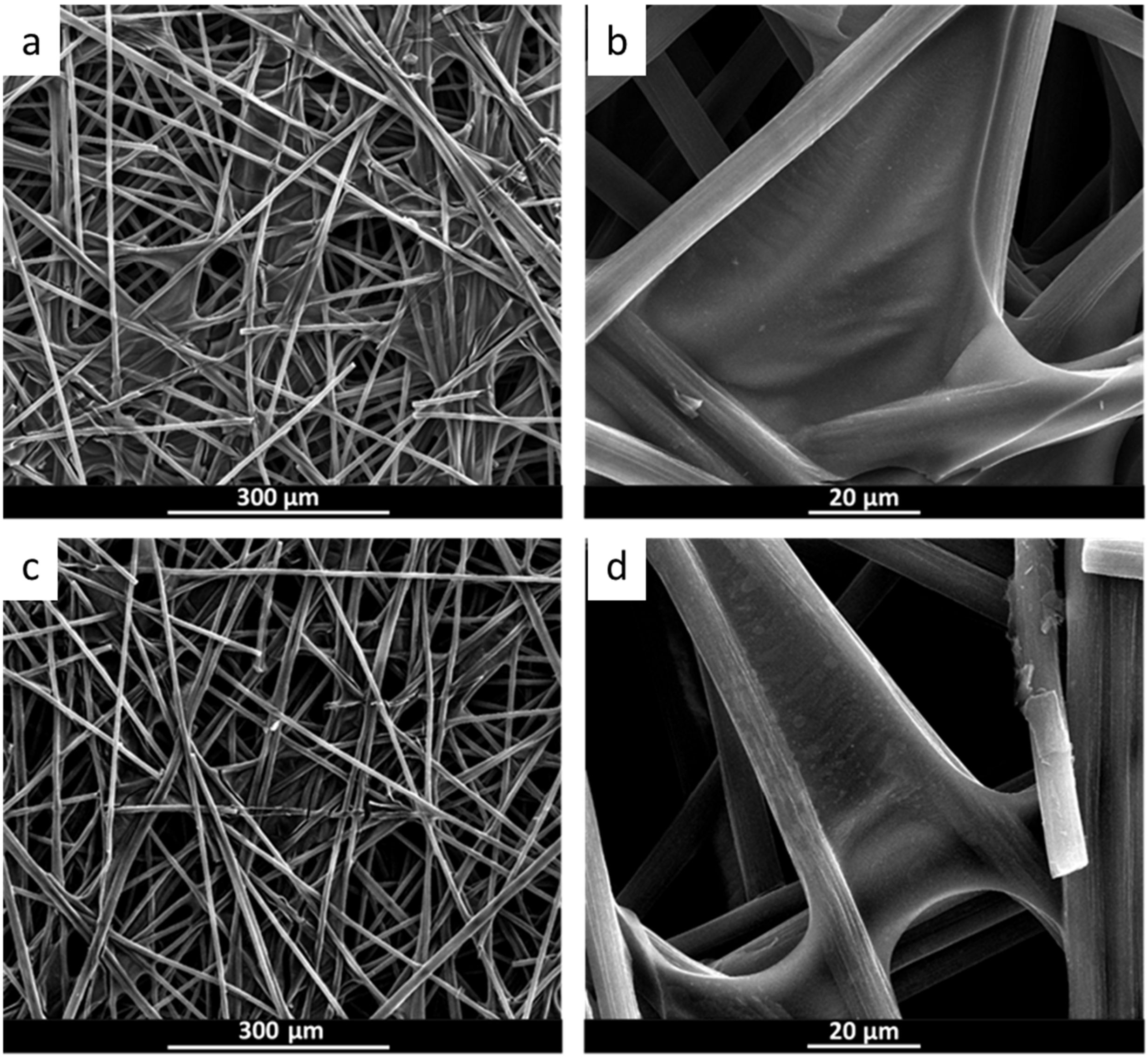

Figure S3. SEM analysis of graphite-based graphene material: a,b) deposited on TCP. c,d) after electrodeposition of NiFe.

c-SEM analysis of pos-catalysis hybrid materials

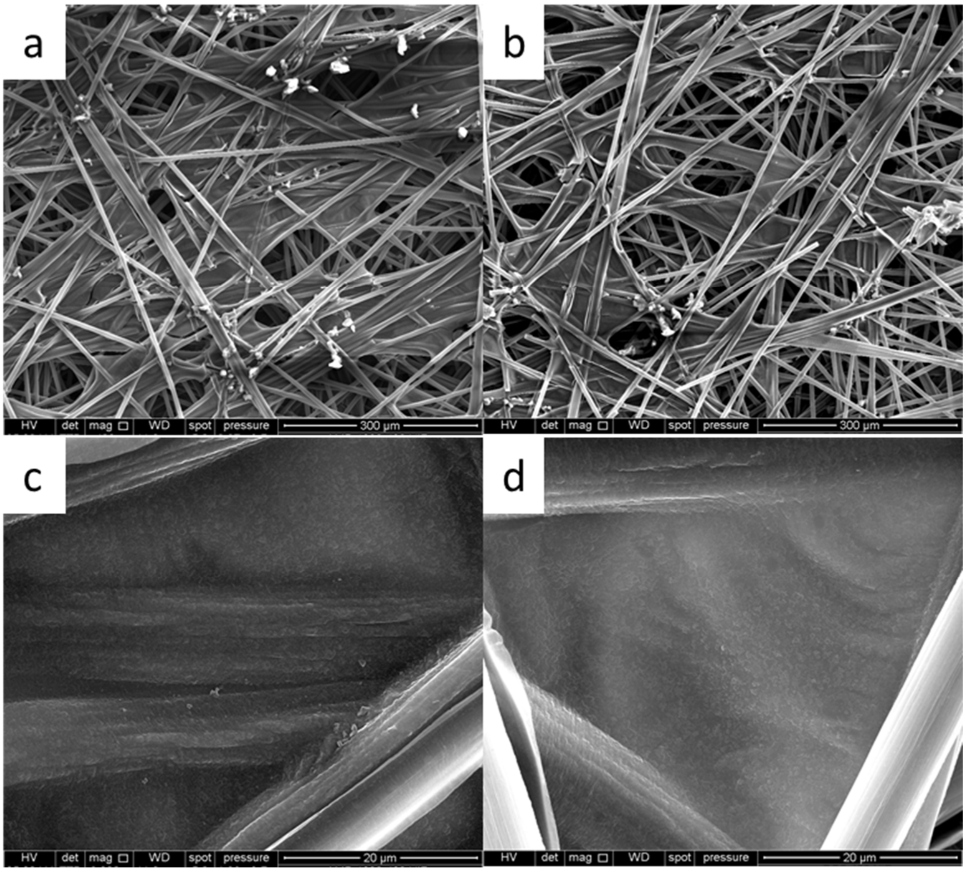

Figure S4. SEM analysis of post-catalysis hybrid electrodes a,c) TCP-Al-GO-NiFe and b,d) TCP-G-GO-NiFe.

3-STEM-EDX analysis of TCP-G-GO-400-NiFe

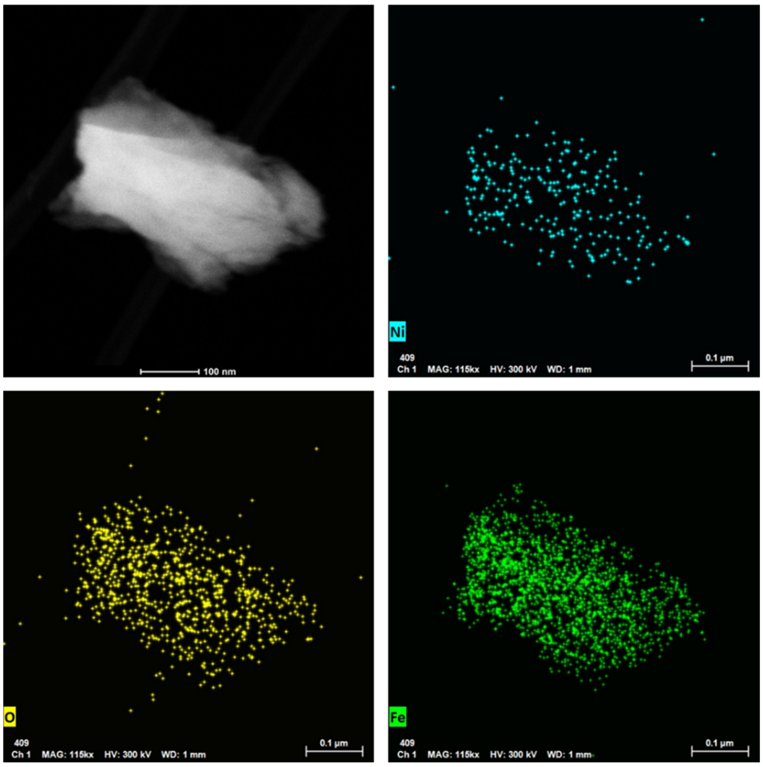

Figure S5. STEM-EDX images and mapping of TCP-G-GO-400-NiFe the homogeneous distributions of elemental Ni, Fe and O along the graphene surface.

4- Electrochemical measurements

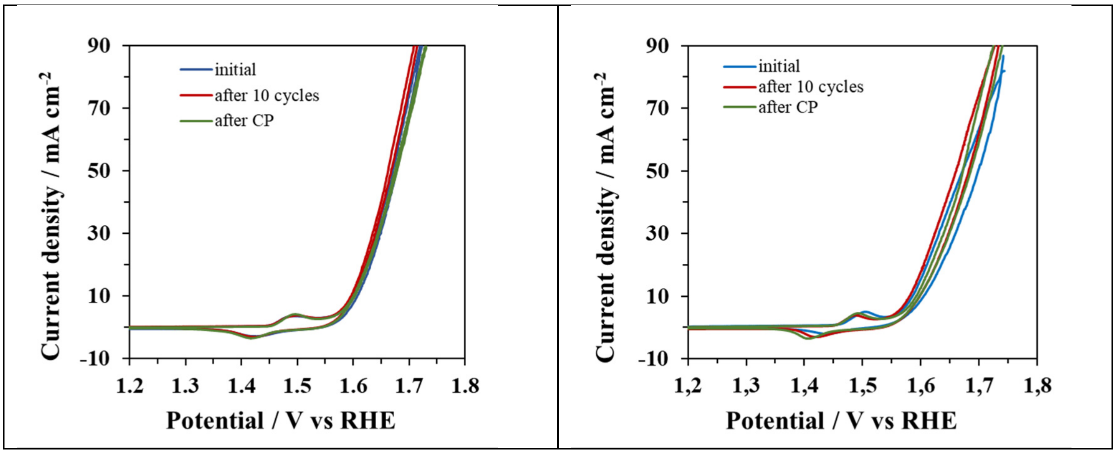

Figure S6. Potential cycling stability results for TCP-G-GO-NiFe (left) and TCP-Al-GO-NiFe (right). All the experiments were carried out at 20 mVs^{-1} in KOH 1M.
